# Replication of Holograms with Corn Syrup by Rubbing

**DOI:** 10.3390/ma5081462

**Published:** 2012-08-21

**Authors:** Nildia Y. Mejias-Brizuela, Arturo Olivares-Pérez, Mauricio Ortiz-Gutiérrez

**Affiliations:** 1 National Institute of Astrophysics, Optics and Electronics, calle Luis Enrique Erro No. 1, Santa María Tonantzintla, Puebla 72840, Mexico; E-Mail: nilyame@inaoep.mx (N.Y.M.-B.); 2 Faculty of Physics and Mathematics, Universidad Michoacana de San Nicolás de Hidalgo, calle Francisco J. Mújica s/n, Col. Felicitas del Río, Morelia 58060, Michoacán, Mexico; E-Mail: mortizg@yahoo.com (M.O.-G.)

**Keywords:** holographic material, biopolymer, computer holograms, replicate holograms

## Abstract

Corn syrup films are used to replicate holograms in order to fabricate micro-structural patterns without the toxins commonly found in photosensitive salts and dyes. We use amplitude and relief masks with lithographic techniques and rubbing techniques in order to transfer holographic information to corn syrup material. Holographic diffraction patterns from holographic gratings and computer Fourier holograms fabricated with corn syrup are shown. We measured the diffraction efficiency parameter in order to characterize the film. The versatility of this material for storage information is promising. Holographic gratings achieved a diffraction efficiency of around 8.4% with an amplitude mask and 36% for a relief mask technique. Preliminary results using corn syrup as an emulsion for replicating holograms are also shown in this work.

## 1. Introduction

Biopolymers, in particular, proteins, have been recently investigated as new materials for holographic registers. Some works based on sugars such as sucrose [1], corn syrup holograms [2], and lactose [3] have realized acceptable diffraction efficiency for holographic applications.

Corn syrup (Karo^®^) is composed of a variety of complex sugars and other compounds in smaller proportions. The main chemical components of any processed honey varieties are fructose and glucose [4]. The fructose and glucose in honey are simple molecules and not chemically bonded as in the sucrose molecule, where they form a 1,2 ortho-glycoside bond.

Humidity limits the investigations of sugars [5], since such compounds are hygroscopic, and therefore, the recording time and the measurement of diffraction efficiency depend on the environmental relative humidity. A registered hologram has a short lifetime as a function of the environmental relative humidity. At a high humidity of around 50% or more, hologram lifetime is in the order of a few days, whereas at a low humidity of around 35% or less the corresponding lifetime is in the order of weeks or months (stored in a locked box free of air moisture). 

As a material for replicating holograms, corn syrup opens new interesting possibilities for constructing holographic devices in the biophotonics field without the toxins present in photosensitive salts or dyes. Some copyrighted works have suggested a route for the replication of micro reliefs for the pharmaceutical industry and food, as described in [5,6]. This work, in addition to an interesting patent on edible holograms [7], manifests the ability of corn syrup for copying holograms by stamping techniques with metal and plastic molds. Such methods are expensive and very technical. The proposed method consists of simple rubbing techniques, which can be used to transfer holographic information to a corn syrup film, and these techniques are easy, cheap, and accessible.

Replication with this material using lithographic and rubbing techniques is also interesting since it is an unusually clean process. The material has an auto developing property, which makes the use of chemical water-based reactive agents unnecessary. The properties contained in corn syrup materials open the possibility of recorder gratings or holograms limited by the pattern mask quality applied to fabricate a holographic pattern. Thus, we decided to investigate corn syrup-like materials for holographic recordings. 

In this research we present the continuation of previous work on corn syrup holograms [2] which showed good attributes for recording holograms. This material was photosensitized with potassium dichromate salt. Due to their toxic character, we decided to generate holographic elements in this material, which were free of photosensitizing agents. This encouraged us to investigate the use of replication techniques through rubbing with amplitude binary masks (Plus X-Pan^®^ photographic film, or amplitude gray levels masks Slavich PFG01^®^) and phase masks (photoresists Shipley 1350J).

Both amplitude and phase masks can replicate holograms and are sensitive to the heat induced by hand rubbing, which can often exceed 50 °C. Anyone can replicate holograms with this technique. One possible application of this material using this technique is to replicate organic periodic structures.

The behavioral regime that this material (corn syrup) follows is of the classic type. Corn syrup does not contain a photosensitizer to induce a physical chemical reaction with the matrix, and simply dries and hardens, copying the relief shape of the pattern used as a mask. We always observe a decline in diffraction efficiency after several days of replication. This decrease is mainly due to the moisture in the environment, since this material is hygroscopic. With protection, this material may last several months, provided the protective film does not degrade.

This work shows the viability of corn syrup as a matrix for holographic elements replication, for the construction and design of diffractive optical devices through computer-generated holograms, and for observing the limitations of this material, which has the benefit of being non-toxic. One of the objectives of this work is to copy holograms at low cost and using only a few technological resources.

## 2. Materials

### 2.1. Corn Syrup

Corn syrup is obtained through the hydrolysis of starch as the end product of sugars like fructose, dextrose, glucose and sucrose [8]. Dextrose is a polymer of a high molecular weight and a starch derivative, which is stored in granules in the endosperm of the kernels of corn seeds. The starch provides the energy required for seed germination. Corn is an abundant source of starch, which comprises around 60% of the total weight of the grain. When fully dry the starch content increases to about 70% of grain weight [9]. High fructose corn syrup (HFCS) is a synthetic monosaccharide with 55% fructose and 45% glucose, and high fructose syrup (HFS) is a synthetic monosaccharide with 42% fructose and 53% dextrose.

In 1965, glucose isomers were discovered through the growth of the microorganisms from the species *Streptomyces*. Once it was possible to grow corn with its body immersed in alcohol to produce a thermostable enzyme in an economically profitable way, corn sugars with a sweetness similar to that of sugarcane became feasible [10,11,12].

Sugars easily hydrate corn syrup owing to their OH groups that can form hydrogen bonds with water molecules, which is the reason why sugars are labeled hygroscopic. The percentage of hydration varies considerably between different sugars [8]. Another important characteristic is that sugars have the capacity to reduce the oxidation of diverse agents, and therefore, these are also called reducing sugars [13].

The viscosity and sweetness of the syrup depends on the extent to which the hydrolysis reaction has been carried out. In order to distinguish different grades of syrup, each is rated according to their dextrose equivalent (DE). DE is a measure of the amount of reducing sugars present in a sugar product, relative to glucose, expressed as a percentage of the natural reduction of water through evaporation and condensation [14]. 

Organic material is suitable for producing diffractive periodic structures, which can stimulate the growth of organic tissues.

### 2.2. Properties of HFS

HFS products on the market are commonly in solid or liquid form. Important properties of these syrups are listed in Table 1.

Syrups containing higher amounts of dextrose may crystallize faster as HFS-42, depending on the temperature of the environment. This crystallization is reversible with the application of heat.

The apparent colors of these products are usually described as clear or white. During the refining process, the syrups are exposed to carbon ion exchange in order to change the tone color of the product [9].

**Table 1:** Properties of high-fructose-syrup [9] *.

Characteristics	HFS-42	HFS-55	HFS-80	HFS-95
Carbohydrates (dry)				
Fructose (%)	42	55	80	95
Dextrose (%)	53	42	18	4
Oligosaccharides (%)	5	3	2	1
pH	4	3.5	3.5	3.5
Refractive index ( at 20 °C)	1.464	1.4786	–	–
Viscosity (Pa s)				
27 °C	0.160	0.760	–	0.575
32 °C	0.100	0.520	–	0.360
38 °C	0.075	0.360	–	0.220
43 °C	0.052	0.240	–	–
49 °C	0.035	0.160	–	–
Density (Kg/cm^3^ at 37 °C)				
Natural	1333.67	1373.21	1383.99	1385.19
Solids	946.63	1056.87	1054.47	1066.45

* With permission of The American Journal of Clinical Nutrition publisher.

### 2.3. Optical Properties of Corn Syrup

#### 2.3.1. Refractive Index

**Figure 1:**
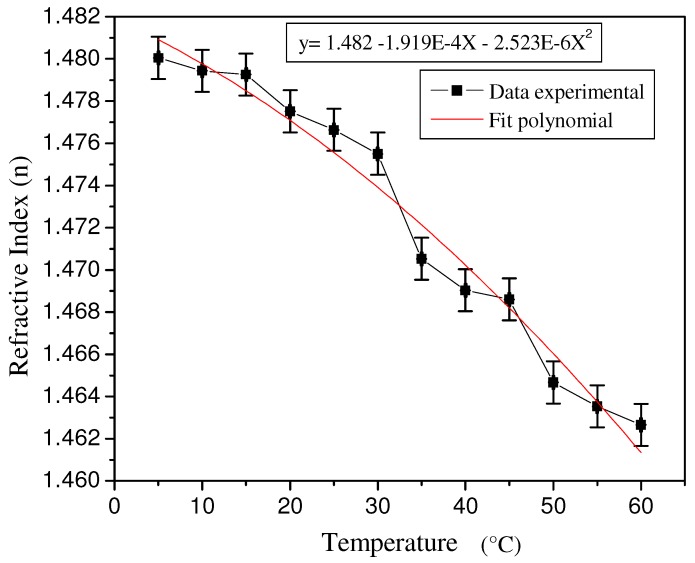
Refractive index of liquid corn syrup as a function of temperature.

The refractive index of corn syrup in liquid form was measured as a function of temperature, as shown in Figure 1. The refractive index was measured using an Abbe refractometer (model Vista C10^®^) using a water bath system for temperature control. Figure 1 shows a decrease in the refractive index value from 1.48 to 1.46 when the temperature is increased from 5 to 60 °C. 

Measurements of the refractive index of a thin layer of dry corn syrup were problematic due to strong chromatic dispersion and were not possible with the Abbe refractometer. 

The data plotted in Figure 1 correspond to an average of four measurements for each point plotted as temperature. The accuracy of the temperature values plotted along the X-axis in the figure was ±0.1 °C. A digital thermometer (Radio Shack^®^ catalog 63-1032) was used for taking the measurement. With reference to the Y-axis in Figure 1, the error in the refractive index, when an Abbe refractometer was used to take measurements, was within ±0.001, and constant for all measurements, based on the data analysis performed for low refractive index readings. 

#### 2.3.2. Absorbance of Corn Syrup

The absorbance profile amplitude shows the spectral response as a function of the wavelength of the UV radiation used. The absorbance was measured with a UV-Vis spectrometer (Perkin Elmer Lambda 3B^®^) with samples deposited on a glass substrate.

**Figure 2:**
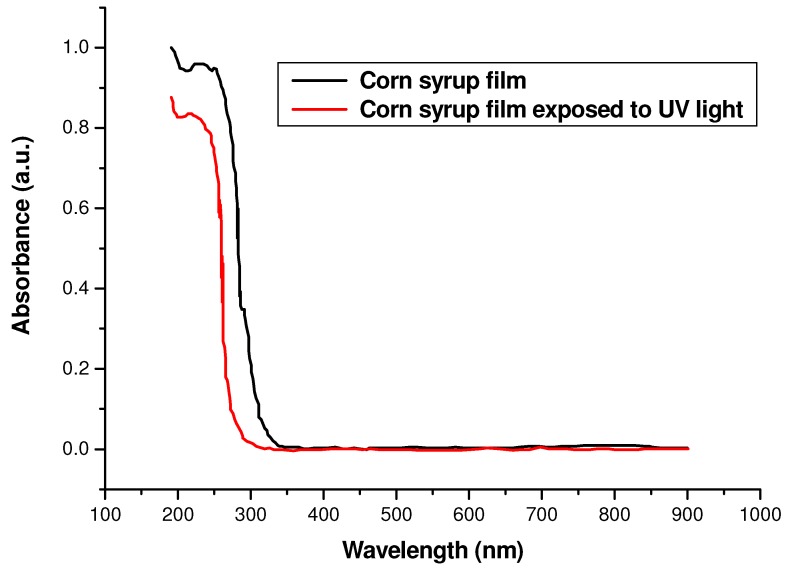
Absorbance versus wavelength, for a dry corn syrup layer of 10 µm thickness.

Figure 2 shows an interesting behavior in the ultraviolet (UV) region between 200 nm and 340 nm for corn syrup material, where high absorbance values were observed. From 350 nm to 900 nm, a flat, zero absorbance was found for the visible region. In other words, this material is completely transparent in the visible range.

However, when this material is irradiated with a UV lamp (Sterilamp^®^, FG4T5) for one minute, a clear difference in absorbance amplitude was observed, presenting a decrease in absorbance with respect to the non irradiated sample.

This behavior indicates that when the sample is irradiated with high-energy (UV) radiation, free radicals are generated, and this is observed as a change in the optical density within the UV region, leading to an amplitude modulation. 

## 3. Methods 

### 3.1. Holograms Replication

Holographic gratings made by light interference can be easily replicated in photoresist using optical replication, either with one-step laser copying or by two-steps holographic copying. For the optical replica technique using photoresist, embossing dies can be made repeatedly, and subsequently, the mass production of exact copies is a relatively simple matter for skilled operators [15,16]. Mechanical replication involves an embossing relief embedded between two protective polymers layers. 

In the present work, we used a soft embossing technique, because the masks used were soft. When rubbed, the mask on the film containing corn syrup transfers the hologram information to the film made with corn syrup. This soft embossing technique is very common; this technique is also used in biophotonic applications [17].

In contrast, simulation programs for generating digital computer hologram [18,19,20,21] based on the Fourier Transform and Fresnel-Kirchhoff diffraction theory can easily transfer a virtual hologram to a photo-sensitive film in order to obtain an amplitude hologram mask. Plus X-Pan^®^ photographic film and/or PFG01^®^ commercial holographic film from Slavich. Lithography was employed for hologram replication [22,23,24] and consisted of the following steps:

(a) Five drops (1 mL) of material (corn syrup) are evenly placed over a clear glass substrate surface (9 cm^2^).
(b) Using a spinner at 600 rpm for 10 s, a thin and uniform film layer is obtained at 23 °C.
(c) Hot air (70 °C) is then immediately applied to the sample for five minutes 23 °C in order to extract a larger quantity of water from the film.
(d) The amplitude (binary or gray levels) or relief mask (hologram) is then placed over the film made with corn syrup, applying light hand rubbing on the mask for one minute for the information to be transferred. This process takes into consideration that the emulsion or the relief of the mask must make contact with the sample.
(e) The mask is finally removed from the corn syrup emulsion. Starting from one corner of the substrate, the mask is removed smoothly and is arched slightly with a uniform motion, until it totally detached.

All steps involved in the replication process were realized at 23 °C and a relative humidity in the 30% to 43% range. The thickness of material after the spinner process was about 7 µm to 10 µm, measured with a digital micrometer (Mitutoyo Corporation^®^ Model IP65). This difference in thickness is usually due to ambient moisture. At low humidity, film thickness is greater, and at high humidity, film thickness is lower.

The amplitude mask has a very small relief due to the development processes; light areas have less relief than dark areas [25]. We also know that by inducing heat by friction in dark areas the temperature is higher than in the light areas [26]. 

Mask information is transferred as density changes of small reliefs and refractive index modulation in located areas of the material. Phase modulation occurs, because the density changes of corn syrup material are proportional to the refractive index and the thickness in the relief used for the mask. Therefore the information is copied onto the corn syrup films as a phase hologram and does not need a developing process as in the wet processes commonly used in photography. Under these conditions, we deduce that the modulation of corn syrup is due to the refractive index and relief modulation. We did not measure the refractive index of a thin layer of dry corn syrup since these films presented strong a chromatic dispersion and could not be measured using the Abbe refractometer. 

As mentioned above, light hand rubbing is used to transfer holographic information onto the corn syrup film and thermal changes are induced during this process. Thermal changes lead to density changes (viscosity), and thereby result in refractive index and relief modulation.

**Figure 3:**
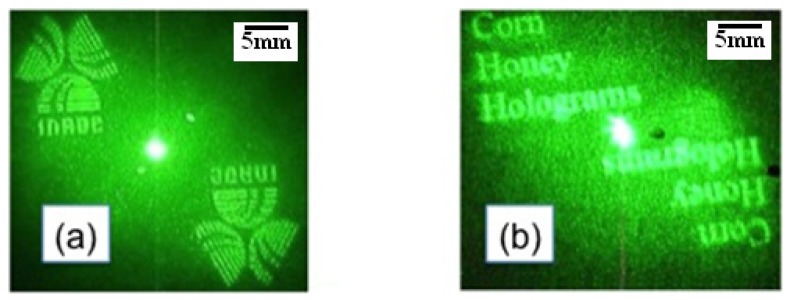
Optical reconstruction of Fourier binary holograms in a corn syrup film (**a**) INAOE logo; (**b**) Hologram of the phrase “corn honey holograms”. The diffracted image of the hologram has a physical size of approximately 3 cm × 3 cm.

Figure 3a shows the optical reconstruction of Fourier holograms using a laser diode at 530 nm (LSR532NL-500). We observed the INAOE logo shown in Figure 3a, and as shown in Figure 3b, we observed the reconstruction of the phrase “corn honey holograms”. Both holograms were replicated with an amplitude binary mask using Plus X-Pan^®^ photographic film. The holograms used to construct these images had a dimension of 5 mm × 5 mm, and contained 1024 × 1024 pixels. By illuminating the hologram (corn syrup) with a divergent spherical wave, the images were reconstructed, as shown in Figure 3a,b. At a distance of 2 m, pictures are taken through a translucent screen. The diffracted image of the hologram is projected onto this screen, which has a physical size of 3 cm × 3 cm. The corn syrup emulsion has an excellent behavior where many different frequencies within the emulsion film are recorded. These results open the possibility for the design of any type of diffraction pattern.

### 3.2. Holographic Grating Replication

**Figure 4:**
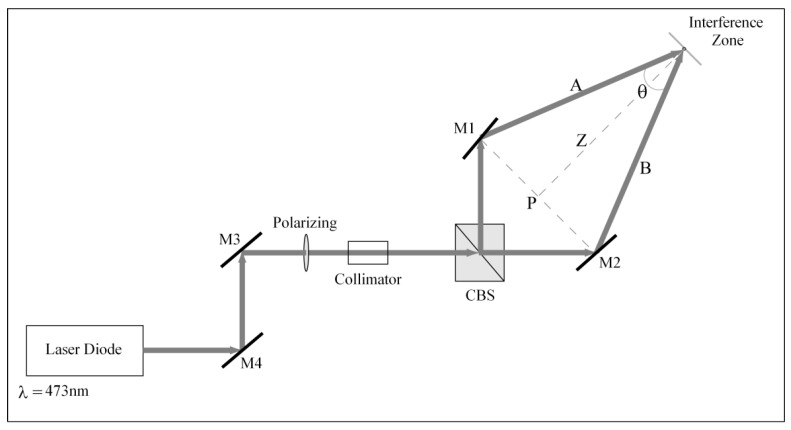
Experimental setup for the recording of holographic gratings in order to construct diffraction gratings with different frequencies and a recording beam at *λ* = 473 nm [30]. *

We used the optical setup shown schematically in Figure 4 to record the amplitude and phase of masks. The symmetrical arms formed by mirror 1 (M1) and mirror 2 (M2) reflected a laser beam into the interference zone where we placed a material for holographic recording of diffraction gratings. The materials we use in this step were photoresist Shypley 1350J^®^ or photosensitive film from Slavich PFG01^®^. With these films, it was possible to construct several diffraction gratings with different spatial frequencies that were used as masks in order to replicate using corn syrup and obtain the module transfer function (MTF) [24,27,28,29].

Figure 4 shows the optical components used to generate an interference zone [30]. We use two different sources to record the holographic gratings. The photoresist Shypley 1350J is photosensitive in blue light and hence, a laser diode is used at 473 nm (LSR473NL-200) with an output power of up to 200 mW. PFG01 film is photosensitive to red light, and hence, a He-Ne laser is used at 632.8 nm with an output of 17 mW. The incident beam on the holographic material is linearly polarized. We ensure successful polarization by using a linear polarizer after alignment mirror 3 (M3) and mirror 4 (M4). Both A and B arms are linearly polarized and form an isosceles triangle between M1, M2 and the interference zone. The main beam is split into two arms using a cube beam splitter prism (CBS). The angle formed between both arms is θ. The incident energy is approximately split into half between each arm. The two beams impinge at a point (interference zone) where an interference pattern is formed. This symmetry allows us to vary the distance Z between the interference zone and point P. Several positions of the interference zone are necessary in order to achieve various angles, which in turn lead to multiple frequencies for holographic gratings. 

The size of the interference zone can be changed by placing a collimator in front of CBS. We worked with an interference area that was approximately 5 mm in diameter. The size of its interface zone also varied, depending on the angle between the arms of the array and the interference zone. 

* With permission of SPIE publisher.

**Figure 5:**
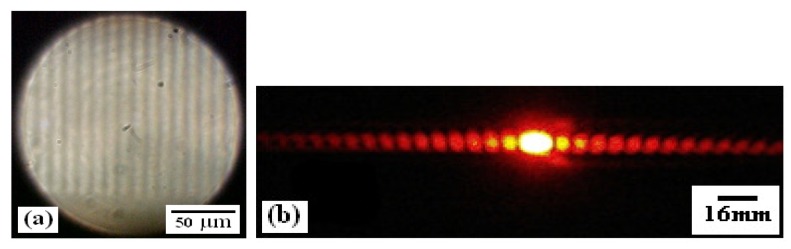
(**a**) Micrograph of a grating recorded on corn syrup film; (**b**) Optical reconstruction of grating transferred to corn syrup.

Twelve holographic grating pattern were transferred to corn syrup material and reconstructed with a wavelength of 632 nm from a He-Ne laser (Melles Griot^®^, 30 mW, λ = 632.8 nm). Figure 5a shows a micrograph of the sinusoidal periodic structure recorded in the corn syrup film with a thickness of about 30 µm. Thickness was measured using a micrometer (Digimatic Model IP65 Mitutoyo^®^). 

The transferred patterns are due to density changes in the corn syrup material, which induce changes in the refractive index (induced by temperature changes by rubbing) and relief (thickness changes). 

Figure 5b also shows a photograph taken at a distance of 25 cm from the hologram (grating) of the symmetrical diffraction pattern from the holographic grating shown in Figure 5a. The behavior of many diffracted orders (Figure 5b) is due to the low spatial frequency of the mask pattern used and by the high contrast of the mask. Ten diffracted orders were measured for each diffraction grating. For this reason, the energy of the first order is low. These diffraction gratings follow the criteria of the Klein-Cook parameter, which corresponds to the regime of Raman-Nath diffraction [31].

#### 3.2.1. Evolution of Diffraction Efficiency by Module Transfer Function 

The module transfer function (MTF) of the material makes the prediction and optimization of the quality of the holographic image [24,27,28,29] possible. The spatial frequency of the imaging system can be described by the MTF [27,28,29]. This parameter is commonly used in optical imaging and determines the ability of the optical system to resolve every detail of the object represented by spatial frequencies. The MTF is a normalized value of the transfer function expressed as a percentage. Similarly, the diffraction efficiency parameter corresponds to a normalization of intensities, namely, the intensity ratio of the +1 diffracted order intensity and the incident beam intensity, which is also expressed as a percentage. The diffraction efficiency of the holographic gratings recorded on photoresist and the PFG01 film was measured with a detector when the grating was illuminated with a laser beam with *λ* = 633 nm. The incident beam was not expanded, and therefore, only a punctuated area of the holographic gratings was reconstructed. Corrections produced by the absorbance and reflectance were not lost. All experimental radiation measurements were realized with a radiometer (Internacional Light, Modelo IL 1700^®^) to collect the total energy of the incident beam and transmitted diffracted beam.

#### 3.2.2. Replication with Photoresist Shipley 1350 J^®^

In this section we present the results of the holographic gratings replication performed using photoresist phase masks (modulated by relief). The technique used to transfer information to the emulsion-sweetened corn syrup is described in Section 3.1.

**Figure 6:**
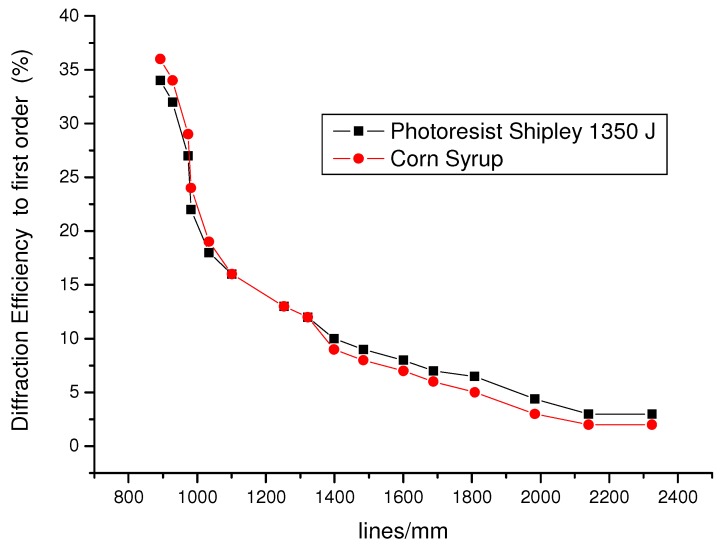
Behavior of spatial frequency modulation from diffraction gratings recorded in positive photoresist Shypley 1350 J^®^ and the replication behavior in corn syrup material.

Figure 6 shows the behavior of the evolution of the diffraction efficiency parameter of holographic diffraction gratings constructed with Shipley 1350 J^®^ photoresist using the symmetrical arrangement of Figure 4. The maximum diffraction efficiency is observed at low frequencies, with a value of around 34%. Values then begin to decline when the gratings are at a higher spatial frequency, reaching a minimum value of 3%.

Furthermore, in Figure 6, we show the diffraction efficiency behavior for diffraction gratings replicated using corn syrup material. The masters for the diffraction gratings were constructed using photoresist. The ability of corn syrup to replicate holograms was excellent. It was observed that for low frequencies, the diffraction efficiency of copying holographic gratings was even higher than that of the same master, which in this case was 36%. This is because the film layer of corn syrup is more transparent compared to the photoresist. However, for high frequencies, the holographic grating diffraction efficiency for replication is less than that for the photoresist master. In general, the replication of the embossed information generated through photoresist with corn syrup films is remarkable. We found nearly the same pattern of behavior for spatial frequency modulation on the MTF of the photoresist. These phase gratings (relief), following the criteria of the Klein-Cook parameter, with Bragg diffraction regime, were 10 μm and 30 μm thick, respectively [31].

#### 3.2.3. Replication with film Slavich PFG01^®^

Replication of holographic gratings using the masks amplitude (modulated by gray levels) was obtained with the Slavich photosensitive holographic film PFG01^®^. 

**Figure 7:**
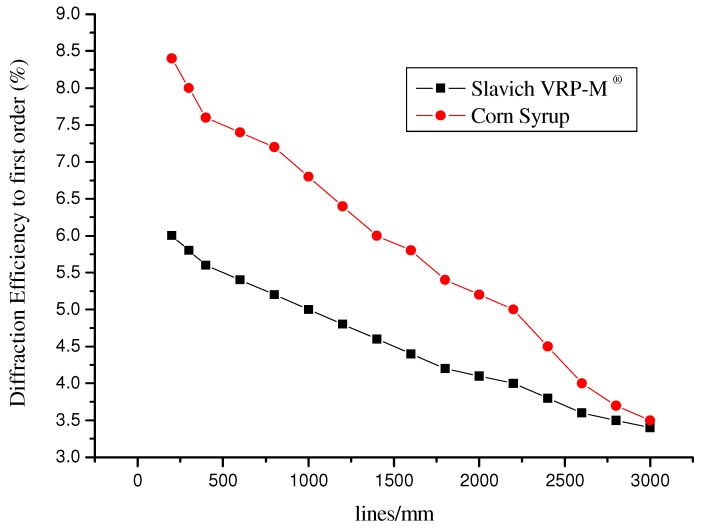
Behavior of spatial frequency modulation from diffraction gratings recorded in the Slavich PFG01^®^ film and replication behavior in corn syrup material.

Figure 7 shows the diffraction efficiency parameter versus spatial frequency of the holographic diffraction gratings constructed with photosensitive film Slavich PFG01^®^ for an arrangement similar to Figure 4, using a wavelength of *λ* = 632.8 nm. The optimal diffraction efficiency in the amplitude mask was observed at short frequencies, with a value of around 6% at the first order peak (congruent with the theory of amplitude holograms). Values then decreased when the gratings were at a higher spatial frequency, reaching a minimum value of 3.4%.

Additionally, diffraction gratings were replicated by the rubbing technique using corn syrup material. The masters of the diffraction gratings were constructed with an amplitude mask Slavich film PFG01^®^. The ability of corn syrup to replicate holograms is excellent. For low frequencies, the diffraction efficiency for copying holographic gratings was higher than that for the master used, which in this case was 8.4%. This change was a result of the fact that the corn syrup film layer was more transparent with respect to the amplitude mask. However, for higher frequencies, the diffraction efficiency for the replication of holographic gratings was less than about 3.5%, but slightly greater than that for the amplitude mask, which was 3.4%.

The values of the spatial frequencies of the interferometer arrangement shown in Figure 3 are different compared to those in Figure 5 and Figure 6 because the two similar experimental arrangements used had different dimensions. One arrangement was designed for the photoresist and the other arrangement was designed for the Slavich photosensitive film.

The thickness of corn syrup is an important parameter that needs to be considered. For phase relief holograms, satisfactory results were obtained for a thin film thickness of about 10 μm or a film thickness in the order of 30 μm or greater. For holograms made with an amplitude mask, satisfactory results were obtained only for film thicknesses of 30 μm or greater. In thin films obtained by a spinner and having a thickness in the order of 10 μm, replications were low in quality. 

When replicating holographic gratings through an amplitude mask using Plus X-Pan^®^ photographic film and/or PFG01^®^ commercial holographic film from Slavich. Corn syrup films with a thickness in the order of 10 µm were required to make a copy of a holographic pattern by the rubbing technique. The corn syrup layer deteriorated rapidly, thereby disabling the replication of the hologram. When using films with a thickness of about 30 µm, holograms were successfully replicated, and the films did not deteriorate. 

These diffraction gratings followed the criteria of the Klein-Cook parameter, which corresponds to the regime of Raman-Nath diffraction. The Bragg regime is attained when a spatial frequency of 400 lines/mm overcome while using a thickness of 30 µm. On the other hand, satisfactory results were not obtained with thicknesses of 10 µm [31]. 

However, the parameter *ρ*, defined as *λ_0_*^2^/ (*Λ*^2^*n_0_n_1_*) defined by Nath, can also be used to determine the type of regime for the phase gratings, *i.e.*, the Bragg or Raman-Nath type. Here, *Λ* is the grating spacing, *λ_0_* is the vacuum wavelength of the light, *n_1_* is the amplitude of the sinusoidal modulation of the refractive index, and *n_0_* is the mean refractive index. The grating thickness does not use *ρ*, and hence, it is not necessary to know the thickness in order to determine which regime is operative [32]. 

#### 3.2.4. Refractive Index with Kogelnik’s Coupled Wave Theory

Experimental observations to indirectly determine the refractive index of corn syrup, for a dry film, using Kogelnik’s coupled wave theory Equation (1) [33].

(1) $$\eta =sin{\left[\frac{\pi d\Delta n}{\lambda cos(\beta )}\right]}^{2}$$

**Figure 8:**
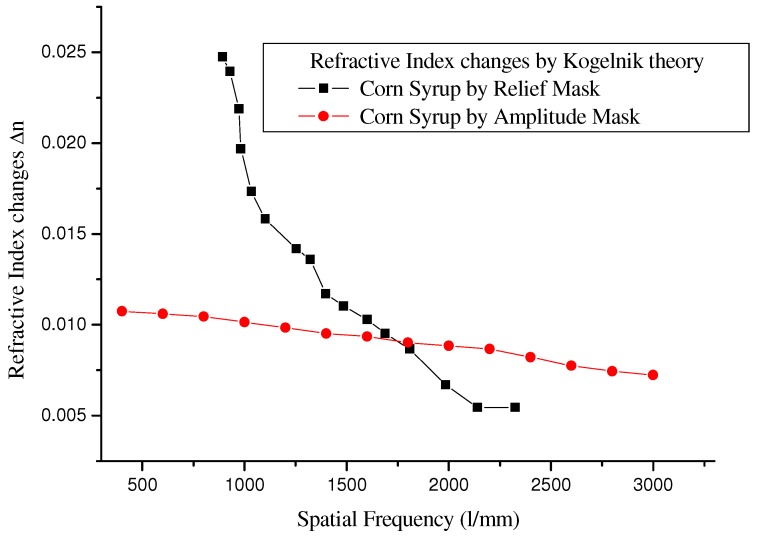
Behavior of spatial frequency *vs.* refractive index changes using Kogelnik’s theory.

Reconstructing gratings at normal incidence, therefore the angle *β* is zero. Solving for Δ*n* as a function of diffraction efficiency Equation (2), and plot with spatial frequencies using the Bragg equation to obtain Figure 8.

(2) $$\Delta n=\frac{{sin}^{-1}\left(\sqrt{\eta }\right)\lambda }{\pi d}$$

Where *η* is the diffraction efficiency of gratings recorded in the corn syrup, *λ* is wavelength of the reading, d the thickness of the sample and *β* is the readout angle inside the photomaterial.

These results were obtained by the diffraction gratings made of corn syrup, with a thickness of 30 μm, using a He-Ne laser *λ* = 632.8 nm, at a normal incidence over the gratings. Measured diffraction efficiencies of gratings led to obtaining values lower than those reported in Figure 6 and Figure 7. This is natural because they were not measured in the Bragg angle.

Figure 8 shows the behavior of the refractive index changes Δ*n*, according to Kogelnik’s theory, as a function of the diffraction efficiency of gratings constructed with corn syrup. With respect to the spatial frequency of gratings, a decrease in the values of the changes in the refractive index Δ*n* was observed for higher frequencies. It also clearly shows the differences in the transfer of information due to the masks used: Masks with relief (phase) show a greater change in refractive index Δ*n*. Hence the diffraction efficiency of gratings has high values. Amplitude masks (binary or gray levels) show a smaller change in refractive index Δ*n*, reflecting a poor diffraction efficiency of the gratings.

## 4. Conclusions

Corn syrup is a promising material for phase (relief) or amplitude hologram replication. Masks can be made using a computer or an optical setup. 

The rubbing technique used in the present work resulted in excellent replication. This technique results in thermal heat dissipation through the mask, in addition to the information contained in the relief mask, and copied material from the corn syrup. The increase in temperature, due to the friction induced by rubbing on the mask material, induces refractive index changes in the corn syrup film. We observed that the refractive index decreased when the temperature increased in corn syrup. For the replication of amplitude holograms, which can be coded with dark and light areas for binary masks, or with gray level masks, it is known that the dark areas are heated more than the light areas in order to induce friction. These thermal differences also contribute to the local modulation of the refractive index.

We found that the ability of corn syrup to replicate holograms is promising compared with gratings recorded on photoresist as observed based on their respective MTF behavior. For low frequencies, the diffraction efficiency for replicating holographic gratings using corn syrup was higher than that for the master. This small change could be associated with the higher transparency of the corn syrup layer with respect to photoresist. However, for high frequencies, the diffraction efficiency of the replicated holographic gratings was less than that for the photoresist master.

The replication of diffraction gratings recorded on a PFG01 film using corn syrup always showed a higher diffraction efficiency than that for an amplitude mask, where a maximum value of 8.4% was achieved for low frequencies.

Figure 6 and Figure 7 show the ability of corn syrup to copy the diffraction patterns with high quality. 

We were also able to determine the appropriate regime for the phase gratings, specifically, the Bragg or Raman-Nath type, using the parameter ρ as defined by Nath. The grating thickness does not used *ρ*, and hence it is not necessary to know the thickness in order to determine the operation regime [32].

We determined indirectly the refractive index by experimental observations for a dry film of the corn syrup using Kogelnik’s coupled wave theory.

Corn syrup has been shown to replicate computer holograms or holograms made with a conventional setup in the laboratory. This material opens new interesting possibilities for constructing holographic devices, e.g., in the bio-photonics field, without the toxins present in conventional photosensitive salts or dyes. Lithographic techniques and a rubbing process were used for replication. These processes are cleaner than usual since corn syrup does not require a developing process. 

This technique can be used to replicate holograms with nontoxic organic materials for biological tests and studies of bio-optics. Laboratories with limited resources or other disciplines can access this technique in order to design periodic elements with textures similar to those of organic tissues.
